# NFκB/IL-6/JAK2/STAT axis in myelofibrosis is a key vulnerability that is targetable and relevant to JAK2 inhibitor treatment resistance

**DOI:** 10.1038/s41408-025-01447-x

**Published:** 2026-01-12

**Authors:** Huiqin Bian, Naseema Gangat, Na Shen, Jiuxia Pang, David Wald, William Tse, Ayalew Tefferi, Fei Yan, Shujun Liu

**Affiliations:** 1 Department of Medicine, The MetroHealth System, Case Western Reserve University, Cleveland, OH USA; 2 Division of Hematology, Mayo Clinic, Rochester, MN USA; 3 Key laboratory of Polymer Ecomaterials, Changchun Institute of Applied Chemistry, Chinese Academy of Sciences, Changchun, China; 4 Department of Pathology, School of Medicine, Case Western Reserve University, Cleveland, OH USA; 5 Gene and Cell Therapy Institute (GCTI), The MetroHealth System, Case Western Reserve University, Cleveland, OH USA; 6 State Key Laboratory of Inorganic Synthesis & Preparative Chemistry, College of Chemistry, Jilin University, Changchun, China

**Keywords:** Cell biology, Myeloproliferative disease

## Abstract

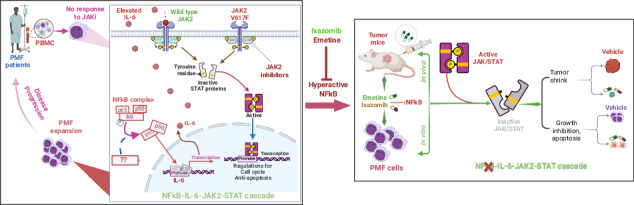

**Dear Editor**,

Myelofibrosis (MF) is a chronic myeloproliferative neoplasm (MPN) characterized by bone marrow (BM) fibrosis, splenomegaly, and ineffective erythropoiesis [1]. Its complex pathogenesis involves constitutional JAK/STAT hyperactivation, elevated cytokines, and hyperactive NFκB [2], though the precise interactions of these pathways are unclear. Current JAK2 inhibitors (JAKi) alleviate symptoms like splenomegaly and improve quality of life but lacks disease-modifying activity, and resistance is common, leading to disease progression [3]. One documented resistance mechanism involves a paradoxical JAK2 hyperphosphorylation upon treatment [4], which prevents dephosphorylation at the Tyr1007/1008 site [5]. However, the precise molecular mechanisms driving this process remain unknown.

In this study, we ran a series of experiments to understand and overcome JAKi treatment resistance in MF. First, we confirmed the phenomenon of paradoxical JAK2 hyperphosphorylation by treating JAK2^V617F^-positive HEL cells [6] with ruxolitinib or BMS-911543. Western blotting and colony analysis revealed that while STAT5 phosphorylation and colony formation are significantly inhibited, JAK2 phosphorylation paradoxically increases (Fig. S1A, B). Further investigation with BMS-911543 demonstrated that this effect involves increased phosphorylation at JAK2-Tyr1008, decreased phosphorylation at Tyr221/570, and concurrent dephosphorylation of STAT5 and AKT (Fig. S1C). To model acquired resistance, HEL cells were serially cultured with escalating concentrations of ruxolitinib or BMS-911543 over eight weeks. Western blotting of the resulting resistant cells consistently showed elevated phosphorylation of JAK2, STAT3, and STAT5 compared to controls (Fig. S1D). Furthermore, BMS-911543 withdrawal from resistant cells induced JAK2 and/or STAT hyperphosphorylation (Fig. S1E), consistent with “cytokine-rebound” effects [5]. These results indicate that JAK2/STAT pathway activation persists not only during acquired resistance but also after drug removal. However, this requires further validation in JAK2^V617F^-negative cells.

To investigate whether active JAK2/STAT signaling predicts patient responses, Western blot analysis was conducted on PBMC from PMF patients participating in clinical trial #NCT01236352 over a 3-month period (Fig. 1A) [7]. As shown in Fig. 1B, JAK2 protein was upregulated in patient #076 with no obvious changes in patients #2534 and #2378. Contrary to prior in vitro and in vivo studies [4, 5], no hyperphosphorylation of loop JAK2-Tyr1008 was observed. Instead, dephosphorylation (#076) or no changes (#2534, #2378) occurred. Furthermore, STAT5 protein expression and phosphorylation increased in #076 and #2534 but decreased in #2378. The lack of correlation between JAK2 and STAT5 protein expression and phosphorylation suggests that regulators other than JAK2^V617F^ contribute to hyperactive STAT5 signaling in PMF. Importantly, clinical data showed that #076 (JAK2^V617F^-negative) shows no improvement in spleen size and #2534 (JAK2^V617F^-positive) has a slightly larger spleen. Both patients displayed increased protein expression and phosphorylation of STAT5. In contrast, #2378 (JAK2^V617F^-positive), who has decreased protein expression and phosphorylation of STAT5, had improved symptoms. Collectively, responsiveness to JAKi such as BMS-911543 largely depends on inactive STAT5 signaling (reduced expression and phosphorylation) but is independent of the JAK2 activity status. This key finding warrants further investigation in larger PMF cohorts.

**Fig. 1:**
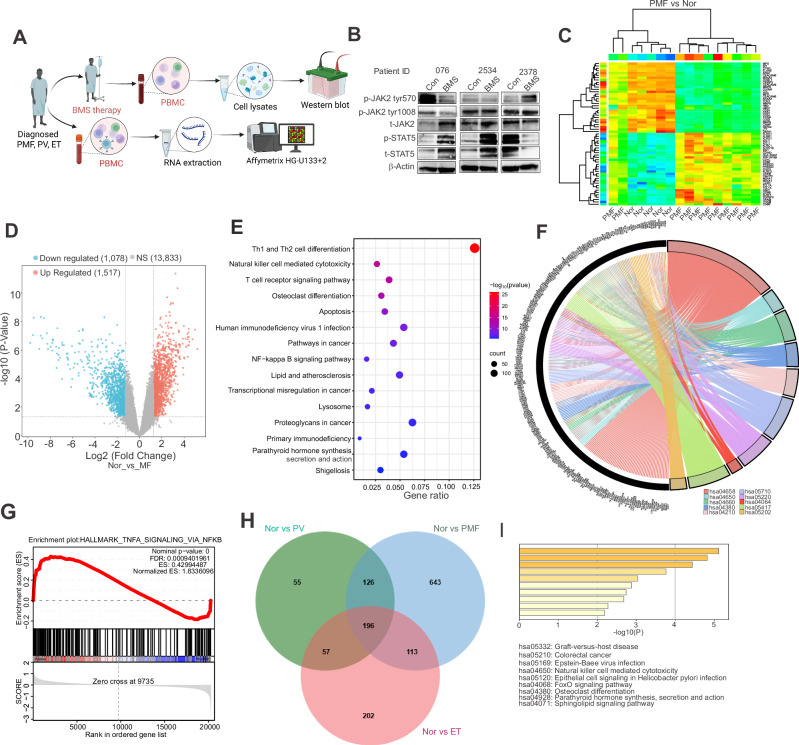
The NFκB signaling pathway and its relevance in myeloproliferative neoplasms. **A** Workflow diagram illustrating the experimental design for analyzing paired pre- and post-treatment samples from PMF patients. **B** Western blot showing changes in the expression and phosphorylation of indicated proteins in PMF patients (*n* = 3) following BMS treatment. **C** Hierarchical clustering heatmap of differentially expressed transcripts in PMF patients compared to healthy donors (HD). Clusters of significantly up- and down-regulated genes are highlighted with black boxes. **D** Volcano plot of gene expression differences between PMF patients and healthy donors. Probes for upregulated genes are shown in red, while probes for downregulated genes are shown in light blue (Log2 FC > 1.25 and FDR-adjusted *P* < 0.05). **E** Bubble chart of enriched KEGG pathways. Upregulated DEGs in HD vs PMF were analyzed to identify the top 10 to 15 most significantly enriched KEGG pathways. The dot size represents the number of DEGs associated with a pathway, while the dot color indicates the adjusted *P*-value (map color keys along with dot size ones are shown on the right). **F** Chord diagram of KEGG pathway enrichment. This diagram shows the relationships between target genes and their enriched KEGG pathways. The most significantly enriched signaling pathways are listed in the bottom-right corner of the diagram, with colors indicating the adjusted *P*-value. **G** GSEA plot showing the enrichment of NFκB-regulated genes in TNFα signaling. The x-axis shows the ranking of genes based on differential expressions (most upregulated to most downregulated), while the y-axis represents the running enrichment score. Expression values are represented as colors and range from red (high), pink (moderate), light blue (low) to dark blue (lowest). **H** Venn diagram illustrating the overlap of upregulated DEGs across 3 comparisons HD vs PV, PMF or ET. The overlapping number represents the shared DEGs among the three comparisons and the non-overlapping numbers specifically show the unique genes in each comparison. **I** Distribution of KEGG pathways for the 196 genes common to all three comparisons shown in **H**. PBMC peripheral blood mononuclear cells, Con control, BMS BMS-911543, DEGs differentially expressed genes, p.adj adjusted *P* values, GSEA Gene Set Enrichment Analysis, GO Gene Ontology, FC fold change, FDR False Discovery Rate, HD healthy donor.

In MF, hyperactive JAK/STAT signaling drives cytokine hypersensitivity and the overproduction of pro-inflammatory cytokines (e.g., IL-6, IL-8), which are linked to a poor prognosis [8, 9]. Our investigation into this pathological process revealed a cytokine-JAK2 feedback loop. Specifically, IL-8 treatment of HEL cells boosted colony formation, consistent with cytokine-potentiated myeloproliferation (Fig. S2A). KEGG analysis of PMF patient cytokine profiles [8, 9] verified active JAK/STAT signaling (Fig. S2B) and Western blotting demonstrated IL-6 or IL-8-induced time-dependent JAK2 hyperphosphorylation (Fig. S2C), confirming this pathological loop. As JAKis don’t fully normalize cytokine levels, targeting cytokine-mediated signaling offers an alternative therapeutic strategy.

IPA analysis of circulating cytokine profiles in PMF patients [9] identified NFκB as a top signaling node (Fig. S2D), indicating non-cell-autonomous activity. To complement this, we investigated cell-autonomous NFκB signaling using RNA microarrays (Fig. 1A) on samples from untreated MPN patients (PMF, *n* = 11; ET, *n* = 6; PV, *n* = 4) and healthy donors (HD, *n* = 5). We identified differentially expressed genes (DEGs) in PMF (*n* = 2673), PV (*n* = 877), ET (*n* = 1272), and between PMFL and PMFH (*n* = 1186) compared to HD (Table S1). These DEGs were visualized in heatmaps (top 65 genes; Fig. 1C, Fig. S3A) and volcano plots (Fig. 1D, Fig. S3B). GO functional enrichment revealed significant terms related to immune cell differentiation and T cell activation (BP), membrane composition (CC), and immune receptor activity (MF) (Fig. S3C). KEGG analysis of HD vs PMF (Fig. 1E, F) and PMFL vs PMLH comparisons (Fig. S3D, E) (padj <0.05) revealed shared upregulated pathways, including Th1/Th2 cell differentiation, NFκB and T cell receptor pathways. NFκB signaling was not significantly altered among downregulated genes (Fig. S3F); however, GSEA revealed NFκB-regulated TNFα signaling as a consistently upregulated hallmark in both HD vs PMF and PMFL vs PMLH (Fig. 1G, Fig. S3G), with core enrichment gene expression patterns correlating with disease stages (Fig. S3H, I). Transcriptomic comparisons of PMF to PV (1520 DEGs) and PMF to ET (2478 DEGs) demonstrated distinct profiles (Table S1). In these later comparisons, KEGG analysis identified NFκB as a top enriched pathway exclusively among downregulated, not upregulated, genes (Fig. S4A, B). Of the 196 genes commonly upregulated across all disease forms (Fig. 1H), NFκB signaling was not significantly enriched (Fig. 1I). RNA microarrays revealed IL-6R upregulation in PMF vs HD (Fig. S5A). Overall, these data indicate distinct transcriptomic profiles and differential NFκB activation across PMF, PV, and ET.

To further explore NFκB hyperactivity in PMF, we used three shRNAs to knock down NFκB expression in HEL cells. Western blotting identified shRNA1 as the most efficient, which was used for subsequent experiments (Fig. S5B). We showed that NFκB knockdown reduces IL-6 expression, whereas NFκB overexpression increases it, demonstrating a regulatory relationship between NFκB and IL-6 (Fig. S5C). Furthermore, NFκB ablation significantly decreased colony formation (Fig. S5D). Collectively, these findings support a cell-autonomous role for NFκB in promoting inflammation and proliferation in PMF.

To complement our genetic ablation studies, we pharmacologically inhibited NFκB in vitro using Ixazomib (MLN9708) and Emetine (Fig. S6A). Western blotting confirmed the dose- and time-dependent proteasome inhibitory activity of Ixazomib via accumulation of polyubiquitinated proteins in HEL cells (Fig. S6B). We also validated that emetine, a known inhibitor of NFκB [10], effectively disrupts NFκB activity in HEL cells. An electrophoretic mobility shift assay (EMSA) using nuclear extracts from emetine-treated HEL cells and ^32^P-labeled DNA probes containing NFκB binding elements revealed that emetine significantly reduces NFκB DNA-binding affinity. A competitive assay demonstrated the specificity of NFκB-DNA interaction, as excess unlabeled probe (50 ×) notably reduced the NFκB-DNA complex (Fig. S6C). Furthermore, MTS and colony-forming assays demonstrated that both emetine and Ixazomib impair cell growth (Fig. S7A) and dose-dependently reduce colony numbers (Fig. S7B). Flow cytometry and Western blotting revealed that both drugs induce apoptosis (Fig. S7C, D) and increase active caspase-3 and caspase-8. Emetine also decreased MCL-1 levels (Fig. S7E–G). These results align with observed NFκB inhibition and impeded PMF cell proliferation.

Based on the promising in vitro results, we investigated the in vivo therapeutic potential of Ixazomib or emetine (Fig. 2A). Subcutaneous xenografts using JAK2^V617F^-positive HEL cells (2 × 10^6^/injection) were established in nude mice. Once tumors (*n* = 6/group) reached ~30 mm^3^, mice were treated subcutaneously with Ixazomib (5 mg/kg), emetine (8 or 16 mg/kg) or vehicle three times weekly for two weeks. As expected, Ixazomib significantly suppressed tumor growth, as evidenced by pronounced reductions in tumor volume (734 ± 133 to 98 ± 14 mm^3^, *P* < 0.001) and weight (582 ± 156 to 87 ± 22 mg, *P* = 0.0005) compared to controls (Fig. 2B–E; Fig. S8A), without reducing mouse body weight (Fig. S8B). Emetine dose-dependently inhibited tumor growth (Fig. 2F, G; Fig. S8C, D) without affecting spleen weight (Fig. S8E). Histological analysis (H&E and IHC Ki-67 staining) revealed that emetine reduces viable tumor cell density and proliferation, as evidenced by lower Ki-67 expression (Fig. 2H). In a prevention study, healthy nude mice were subcutaneously treated with emetine (16 mg/kg) three times weekly for two weeks before injecting HEL cells into both flanks. Despite 100% tumor incidence (*n* = 8 tumors/group), emetine significantly inhibited tumor development, resulting in markedly smaller and softer tumors that are barely sufficient for subsequent histological analysis (Fig. 2J–L). Our results suggest that these agents may be considered as alternative therapeutic options for PMF patients exhibiting hyperactive NFκB signaling. Further validation in leukemic and PDX mouse models is warranted.

**Fig. 2:**
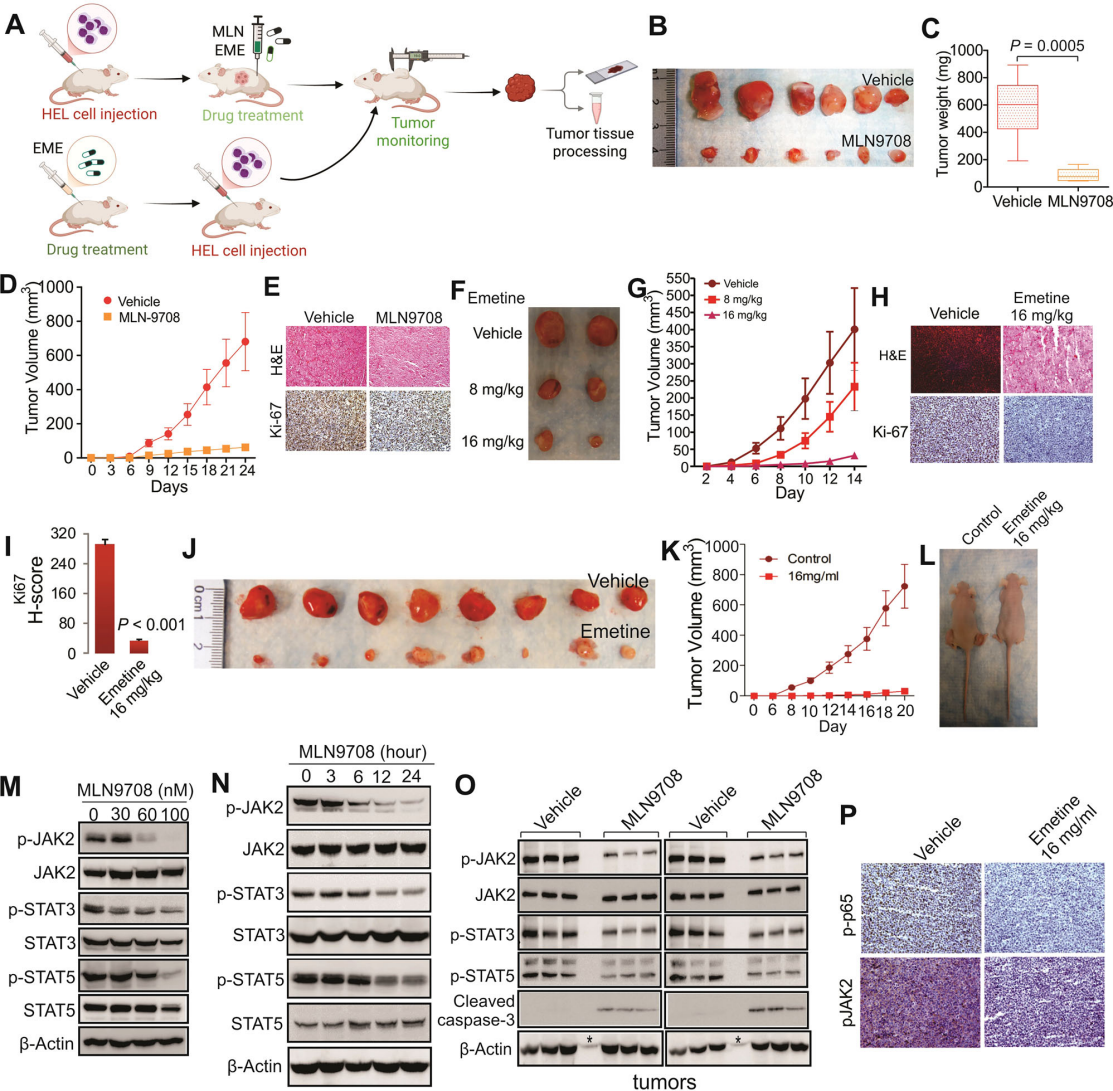
NFкB inhibition suppresses PMF cell proliferation and survival via JAK2-STAT signaling in both in vitro and in vivo models. **A** Workflow diagram illustrates the establishment and characterization of HEL xenograft tumor-bearing nude mice receiving either Ixazomib, emetine or a vehicle control. **B** Representative photographs of tumor volume and weight from HEL xenograft-bearing mice following treatment with Ixazomib or vehicle (*n* = 3 tumors/group). **C** Quantification of tumor weight from the xenograft experiments (*n* = 6 tumors/group). **D** Quantification of tumor volume measured over time (*n* = 6 tumors/group). **E** Histological analysis of tumor tissue. Representative images are shown for H&E staining and Ki-67 antibody staining (*n* = 3). **F** Representative images of xenograft tumors from mice bearing HEL cell xenografts treated with emetine or vehicle (*n* = 6 tumors/group). **G** Quantification of tumor volume from (**F**). **H** Representative images of H&E and IHC (Ki-67) staining of tumor sections. **I** Quantification of Ki-67 signals shows decreased proliferation with treatment. **J**–**L** Protective effect of pre-treating nude mice with emetine before injection with HEL cells. **J** Visual analysis of tumors, (n = 8 tumors/group) **K** Quantification of tumor volume overtime and **L** representative images of tumor-bearing mice. **M**, **N** HEL cells were treated with Ixazomib for 48 h and the whole cell lysates were subjected to Western blot for changes in JAK/STAT pathway. **O** Western blot analysis of whole-protein lysates from excised tumors demonstrates changes in the JAK/STAT pathway. *Indicates a non-specific band. **P** Representative images (*n* = 3) of IHC staining in HEL xenograft tumors. All data are presented as mean ± SD. The results in Western blot represent three independent experiments. H&E Hematoxylin and eosin, IHC immunohistochemistry staining, MLN MLN9708 Ixazomib, EME Emetine.

We investigate how Ixazomib and emetine suppress proliferation in JAK2^V617F^-positive HEL cells and tumors by assessing NFκB activity. Both drugs significantly decreased NFκB phosphorylation at Ser536 (an indicator of NFκB activation [11]) in HEL cells (time- and dose-dependently via Western blotting; Fig. S9A) and tumor tissues (via IHC; Fig. S9B), confirming their function as NFκB inhibitors. Given JAK2’s role in cytokine signaling [12] and NFκB’s role in pro-inflammatory cytokine synthesis, we hypothesized that this inhibition disrupts the IL-6/IL-8-JAK2-STAT cascade, a pathway implicated in PMF [8, 9]. In HEL cells, Western blotting revealed that Ixazomib reduced IL-6 and IL-6R levels (Fig. S9C). Furthermore, both compounds effectively inhibited the phosphorylation (but not total protein) of key signaling components, JAK2, STAT5, STAT3 and NFκB, both in vitro (HEL cells) (Fig. 2M, N; Fig. S9D, E) and in vivo (HEL tumor-bearing mice) (Fig. 2O, P; Fig. S9F). Further evidence came from NFκB knockdown, which mimicked the treatment effect through dephosphorylating JAK2 (Fig. S9G). Additionally, Ixazomib induced apoptosis in tumors, increasing caspase-3 levels (see Fig. 2O). Our data therefore link the inhibition of the NFκB-IL-6/IL-8-JAK2-STAT cascade to the anti-proliferative actions of Ixazomib and emetine. Overall, we describe a key signaling axis in PMF, demonstrating that the disease’s characteristic hyperactive STAT signaling functions independently of JAK2 activity and phosphorylation.

## Supplementary information

Supplementary Materials

Dataset 1

## Data Availability

All raw data not present in the manuscript are available from the corresponding author upon reasonable request.
