# Supplementary material for: NFκB/IL-6/JAK2/STAT axis in myelofibrosis is a key vulnerability that is targetable and relevant to JAK2 inhibitor treatment resistance
Source: Blood Cancer J. 2026 Jan 12;16(1):13. doi: 10.1038/s41408-025-01447-x (PMC12795839; doi:10.1038/s41408-025-01447-x)
Supplement: Supplementary file 1 — Supplementary Materials [file 41408_2025_1447_MOESM1_ESM.docx]

**Supplementary Material**

**NFκB/IL-6/JAK2/STAT axis in myelofibrosis is a key vulnerability that is targetable and relevant to JAK2 inhibitor treatment resistance**

Huiqin Bian, Naseema Gangat, Na Shen, Jiuxia Pang, David Wald, William Tse, Ayalew Tefferi, Fei Yan, Shujun Liu

- **Supplemental Figures and Legends 1-9**
- **Supplemental Methods**
- **Supplemental References**

**Suppl. Figure 1. Alterations in JAK/STAT signaling in response to JAK2 inhibitor treatment. A** HEL cells were treated with various doses of ruxolitinib for 3 hours. Western blot was used to determine changes in STAT and JAK proteins. **B** HEL cells were exposed to various concentrations of ruxolitinib for 6 hours and subjected to colony-forming assays. The graph indicates the colony number from three biological replicates, mean ±SD. **P* <0.05, ***P* <0.01, ****P* <0.0001. **C** HEL cells were treated with various doses of BMS-911543 for 24 hours. The total cell lysates were subjected to Western blot for changes in STAT, JAK and AKT proteins. **D**, **E** HEL ruxolitinib- or BMS-911543-resistant or sensitive cells were growing in drug free medium for 48 hours, and the total cell lysates were subjected to Western blot for changes in JAK and STAT proteins. The data in Western blot represents three independent experiments. p, phosphorylation; t, total.

**Suppl. Figure 2. Elevated cytokine levels induce the activation of NFкB and JAK/STAT signaling.** **A** HEL cells were treated with 1 ng/ml IL-8 protein for 24 hours (h) and subjected to colony-forming assays in medium supplemented with IL-6 protein. The graph indicates the colony number from three biological replicates, mean ±SD. **P* <0.05. **B** Distribution of KEGG pathways based on cytokine overproduction in MNP patients. The distribution of the fold enrichment levels of terms for diverse molecular functions categories is presented. **C** HEL cells were treated with 1 ng/ml IL-6 (left) or IL-8 (right) protein for the indicated time points and subjected to Western blot analysis. The data represents three independent experiments. **D** The top networks identified by Ingenuity Pathway Analysis (IPA). The function of the top network is involved in NFкB signaling. A straight line indicates a direct interaction; a dashed line indicates indirect interaction.

**Suppl. Figure 3. Differentially expressed genes and pathways identified in patients with ET, PV or PMF compared to healthy donors. A** Heat maps for the differentially expressed genes (DEGs). Each column represents PB sample, and each row represents a single gene. Colors represent changes in gene expression, from upregulated (red) to downregulated (green). **B** The volcano plot shows significantly changed genes (FDR <0.05, log2 FC ≥1.25). **C** Go enrichment analysis of top 10 enriched GO terms in BP, CC and MF. **D** Bubble plot of the KEGG enrichment pathways of upregulated DEGs in PMFL vs PMFH. A higher gene ratio represents a higher level of enrichment. The size of the dot indicates the number of target genes in the pathway, and the color of the dot reflects the *P*-value range. **E** Chord diagram of KEGG pathway showing relationships among target genes, annotation, and enrichment. Top KEGG signaling pathways are listed in the bottom right corner of the chord diagram. **F** Distribution of KEGG based on the downregulated DEGs in HD vs PMF. The distribution of fold enrichment levels for various molecular function categories is presented. **G** GSEA identified the NFкB-regulated gene set in TNFα signaling pathways as a common hallmark in PMFL vs PMFH. On the x-axis, genes are ranked from the most up-regulated (left) to the most down-regulated (right) between groups. **H**, **I** Heat maps for core enrichment genes regulated by NFкB within TNFα signaling in HD vs PMF (**H**) or PMFL vs PMFH (**I**). Expression values are represented as colors ranging from red (high), pink (moderate), light blue (low) to dark blue (lowest).

FC, fold change; BP, biological process; CC, cellular component; MF, molecular function.

**Suppl. Figure 4. Differential pathway activation and repression revealed by RNA sequencing in PMF compared to PV or ET patients. A**, **B** Distribution of KEGG based on the up- or down-regulated DEGs in PMF vs PV (**A**) or ET (**B**). The distribution of the fold enrichment levels of terms for diverse molecular function categories is presented.


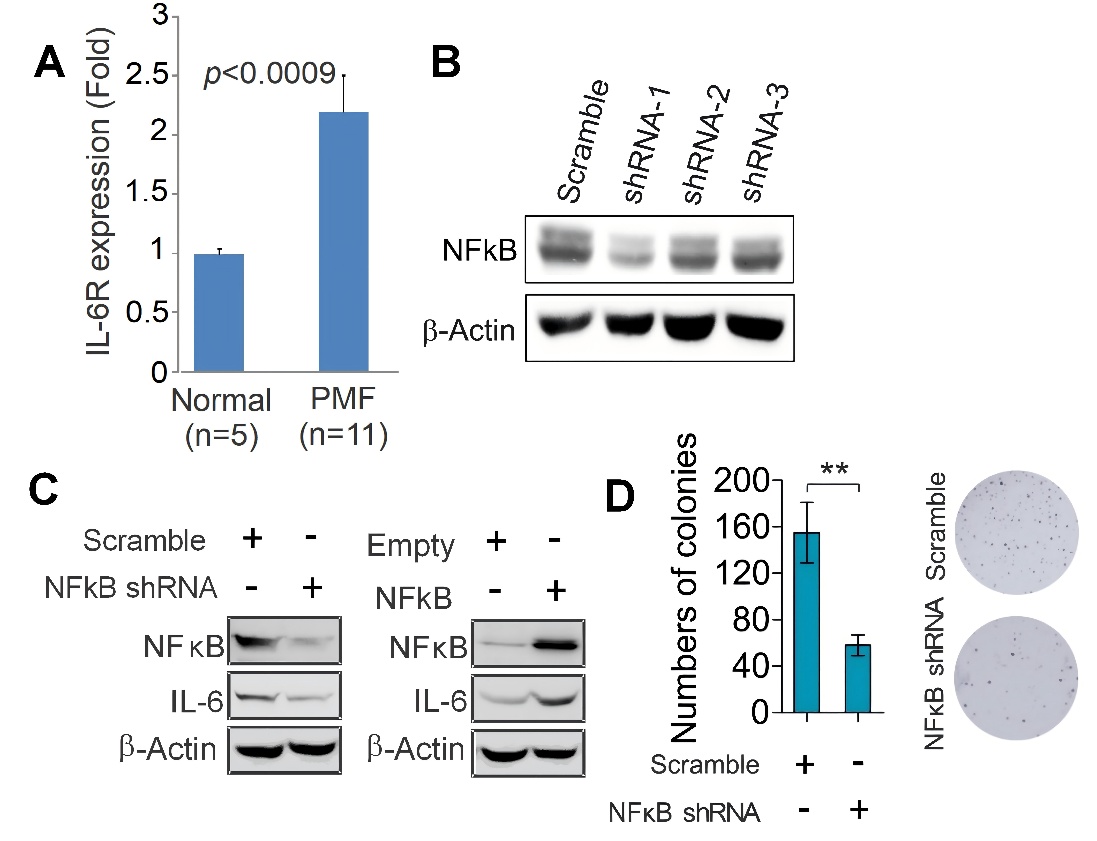


**Suppl. Figure 5. NFκB inactivation suppresses proliferation of PMF cells. A** Relative expression of IL-6R detected by RNA microarrays in PMF patients vs healthy donors. **B** HEL cells were transfected with scramble or three shRNA vectors against NFκB, and the alteration of NFκB expression was assessed by Western blot. shRNA1 showed the best efficacy of NFκB knockdown and was selected for further investigation. **C** HEL cells were transfected with an NFκB expression or shRNA vector for 48 hours, and Western blot was employed to assess the change in IL-6 levels. **D** HEL cells were transfected with NFκB, and cell proliferation was determined by colony-forming assays. The graph indicates the colony number from three biological replicates, mean ±SD. ***P* <0.01.

**Suppl. Figure 6. The inhibitory effects of MLN9708 or emetine on proteasome or NFкB activity in PMF cells. A** The external view of *Psychotria ipecacuanha* or *Cephaelis ipecacuanha*, from which emetine was extracted, and the chemical structure of emetine. **B** HEL cells were treated with different doses of Ixazomib for 24 hours (upper) or 60 nM of Ixazomib for 0, 3, 6, 12, and 24 hours (lower). The whole lysates were subjected to Western blot analysis for the poly-ubiquitinated proteins. **C** EMSA assays for NFкB DNA binding affinity. The nuclear proteins from HEL cells treated with different doses of emetine for 24 hours were incubated with ^32^P-labeled probe containing NFκB binding sites. The specificity of DNA binding was demonstrated by the reduction of complexes with excess (50 ×) unlabeled NFκB consensus binding site (lane 7) probes. Note: MLN9708 is Ixazomib; the β-actin bands are shared between **B** (upper panel) and Fig. 2M, and between **B** (lower panel) and Fig. 2N, as these data were from the same experiment.

**Supplementary Figure 7. Treatment with MLN9708 or emetine inhibits PMF cell growth. A** HEL cells were exposed to various concentrations of MLN9708 for 24 or 48 hours and subjected to MTS assays. Shown are the mean values ±SD of n = 3 independent experiments with quadruplicates. **B** HEL cells were exposed to various concentrations of emetine for 6 hours and subjected to colony-forming assays. The graph indicates the colony number from three biological replicates, mean ±SD. **P* <0.05. **C**, **D** HEL cells were treated with MLN9708 (**C**) or emetine (**D**) for 24 hours, and the cell apoptosis was assessed by flow cytometry. **E**-**G** HEL cells were treated with MLN9708 (**E**, **F**) or emetine (**G**) for 24 hours, and the levels of caspase were determined by Western blot. The data represents three independent experiments.

**Suppl. Figure 8. Administration of MLN9708 or emetine inhibits PMF tumor cell growth.** About 2 × 10^6^ of HEL cells were injected subcutaneously into both left and right flank of nude mice (4-6 weeks old). When the tumor size approached 30 mm^3^, Ixazomib or emetine was administered subcutaneously. **A** Visual analysis of tumor-bearing nude mice in the HEL xenograft model (n=3 mice/group) upon Ixazomib or vehicle treatment. **B** Graph showing the changes in the body weight of the tumor-bearing mice. **C** Visual analysis of tumor-bearing mice in the HEL xenograft model (n = 2 mice/group) treated with emetine or vehicles. **D**, **E** Graphs showing the changes in tumor (**D**) or spleen (**E**) weight of the tumor-bearing mice (n = 4 tumors/group). Data is means ±SD, **P* <0.05.

**Suppl. Figure 9.** **Ixazomib or emetine impairs** **NFкB/JAK2/STAT signaling.** **A** HEL cells were treated with various doses of Ixazomib for 24 hours (upper panel) or with a fixed concentration of 60 nM Ixazomib for indicated time points (lower panel), and whole cell lysates were subjected to Western blot for NFкB protein and phosphorylation. **B** Bars represent the quantification of IHC staining using a p-p65 antibody for sections of emetine-treated tumors (n=3 tumors/group). **C** HEL cells were treated with various doses of Ixazomib for 24 hours (upper panel) or with a fixed concentration of 60 nM Ixazomib for indicated time points (lower panel), and whole cell lysates were subjected to Western blot for the expression of IL-6 and IL-6R. **D**, **E** HEL cells were treated with emetine and the whole cell lysates were subjected to Western blot for JAK2, NFкB, STAT and MCL-1. **F** Bars represent the quantification of IHC staining using a p-p65 antibody for sections of emetine-treated tumors (n=3 tumors/group). **G** HEL cells were transfected with scramble or NFкB shRNA vectors, and the alteration of NFкB and JAK2 was assessed by Western blotting.

**Supplementary Methods**

**Plasmids, shRNA and reagents**

Human NFкB (#21966) plasmids were purchased from Addgene. Three shRNAs and their control vectors against NFкB were obtained from the genomic center at University of Minnesota. Ixazomib (MLN9708, Cat# S2181) or ruxolitinib (Cat# S1378) were purchased from Selleck Chemicals, but BMS-911543 (Cat# 21088) from Cayman Chemicals, and dissolved in ethanol for pre-clinical tests and in DMSO for cell culture experiments. IL-6 (Cat# 206-IL) and IL-8 (Cat# 208-IL) recombinant proteins were from the R&D system. Emetine (Cat# SMB01061) was from Sigma Aldrich.

**Cell culture and transfection**

HEL cell line carrying JAK2 mutation (Cat# TIB-180) was newly purchased from American Type Culture Collection and grown in RPMI 1640 (GE Healthcare Cat# SH30027.01) supplemented with 10% fetal bovine serum (FBS, Gibco by Life TechnologiesTM Cat# 16140-071) and Antibiotic-Antimycotic (Gibco by Life TechnologiesTM Cat# 15240062) at 37°C under 5% CO_2_. No cell line used in this paper is listed in the database of commonly misidentified cell lines maintained by ICLAC (International Cell Line Authentication Committee). The introduction of plasmids and shRNAs into HEL cells was performed as described previously.[[1](#_ENREF_1),[2](#_ENREF_2)] Briefly, 1 × 10^7^ HEL cells were resuspended in 380 μl serum- and antibiotic-free medium, mixed with 15 μg expression or shRNA and the relevant vehicle vectors in a 0.4 cm gap sterile electroporation cuvette and electroporated using Gene Pulser Xcell electroporation system (Bio-Rad, Cat# 165-2660), according to the manufacturer’s instruction.

***In vitro* establishment of resistant HEL cells**

HEL cells were subjected to a dose-escalation strategy to generate resistant lines. Initially, cells were passaged in medium containing a low concentration (0.1 µM) of either ruxolitinib or BMS-911543. The concentrations of these drugs were sequentially increased (to 0.3 µM and 1 µM) over a 6–8-week culture period. Cells cultured in parallel without drug served as the parental negative controls. Cells were considered resistant once they could routinely grow in medium containing 1 µM ruxolitinib or BMS-911543.

**Cell proliferation and apoptosis assays**

Cell growth was determined by the MTS assay using the CellTiter 96^®^ Aqueous One Solution Cell Proliferation Assay kit (Promega, Cat# G8741). In brief, 2000 cells were seeded in a 96-well microplate. After 24-48 hours treatment, 20 µl of MTS solution was added to each well for 2-4 hours at 37°C. The viable cells were detected by reading the absorbance of the metabolized MTS at a wavelength of 490 nm using the BioTek microplate reader.

Cell apoptosis was assessed using the Annexin V-PI Apoptosis Detection Kit I (BD PharmingenTM, Cat# 556547) according to the manufacturer’s instructions. Samples were then analyzed by flow cytometry, and data processing was performed using FlowJo software (Version 7.6.1)

**Hematoxylin and eosin (H&E) and immunohistochemistry (IHC) staining**

Tumors and tissues collected from our animal studies were fixed in 4% paraformaldehyde/PBS. The paraffin-embedded samples were cut to 7 μm thickness and stained with Hematoxylin and Eosin (H&E). All section slides were then microwaved in 10 mM citric acid buffer (pH 6.0) at 70% power for 10 min to unmask antigens. Endogenous peroxidase was quenched using 3% hydrogen peroxide for 20 min followed by rinsing with PBS. Nonspecific binding was blocked with 10% goat serum for 40 min, then with avidin and biotin (Vector Laboratories) for 15 min each. Primary antibodies against Ki-67 (1:500, Abcam, Cat# ab16667), phospho-JAK2 (1:500, Cell Signaling, Cat#3771), and phosphor-NFkB p65 (1:500, Cell Signaling, Cat# 3033) were incubated at room temperature for 1 hour, respectively. For detection of primary antibodies, the Vectastain Elite ABC-peroxidase Rabbit or Mouse IgG Kit (Vector Laboratories) was used. Samples were developed with 3, 3′-diaminobenzidine (Vector Laboratories), counterstained with hematoxylin, and mounted. Stained slides were viewed and photographed with a Leica microscope mounted with a high-resolution spot camera, which is interfaced with a computer loaded with Image-Pro Plus software.

**Clonogenic assays**

Methylcellulose colony formation assays were performed in MethoCult® GF H4434 (Stem Cell Technologies, Cat# 04330) according to the manufacturer’s instructions. Briefly, six hours post-transfection or drug exposure, cells were harvested and diluted in Iscove’s Modified Dulbecco's Medium (IMDM) supplemented with 2% Fetal Bovine Serum (FBS) to a concentration of 5000 - 10000 cells/ml (10 × the final concentration). A 0.3 mL aliquot of this cell suspension was thoroughly mixed with 3.0 mL of MethoCult medium and dispensed into a 35 mm culture dish. Colonies (>125 μm) were scored 7-10 days later.

**Western blotting**

After the various treatments, the whole cellular lysates were prepared by harvesting the cells in 1 × cell lysis buffer [20mM HEPES (pH 7.6), 150 mM NaCl and 0.1% NP40] supplemented with 1 mM phenylmethane sulfonyl fluoride (PMSF, Sigma, Cat# 10837091001), 1 × Phosphatase Inhibitor Cocktail 2 and 3 (Sigma, Cat# P5726, P0044), and 1 × protease inhibitors (protease inhibitor cocktail set III, Calbiochem-Novabiochem, Cat# 539134). The Western blot was performed as previously described.[[1](#_ENREF_1),[2](#_ENREF_2)] Briefly, the proteins were resolved by sodium dodecyl sulfate (SDS)–polyacrylamide gel electrophoresis, transferred onto PVDF membranes (GE Healthcare, Cat# 10600023), blocked by 5% non-fat milk followed by probing with first and HRP-conjugated secondary antibodies, such as goat anti-rabbit IgG (Cell Signaling, Cat# 7074S), horse anti-mouse IgG (Cell Signaling, Cat# 7076S), rabbit anti-goat IgG (Invitrogen, Cat# 31402). The primary antibodies used were IL-6 (Abcam, Cat# ab6672), IL-8 (Abcam, Cat# ab18672), β-Actin (Santa Cruz, Cat# sc-47778), NFкB-p65 (Santa Cruz, Cat# sc-372); caspase-3 (Cell Signaling, Cat# 9662), caspase-8 (Cell Signaling, Cat# 4790), STAT3 (Cell Signaling, Cat# 4904), STAT5 (Cell Signaling, Cat# 9363), JAK2 (Cell Signaling, Cat# 3773), phospho-STAT3 (Tyr705) (Cell Signaling, Cat# 9131), phospho-STAT5 (Cell Signaling, Cat# 9351) and phosphor-JAK2 (Tyr 1007/1008) (Cell Signaling, Cat# 3771).

**Electrophoretic mobility-shift (EMSA) assays**

Three pairs of oligonucleotides containing putative NFκB-binding sites were chemically synthesized, and complementary oligos were annealed and labeled with ^32^P-dCTP and Klenow. All reactions were processed on ice except for those indicated. Total protein extracts were isolated from HEL cells using M-PER (Mammalian Protein Extraction Reagent, Thermo Fisher Scientific, Cat# 78503), and nuclear extracts were prepared using NE-PER (Nuclear and Cytoplasmic Extraction Reagent, Pierce, Cat# 78833). EMSA was performed with total extracts or nuclear extracts and NFkB binding elements by ^32^P-labeled using 5’ end-labeling methods.

**RNA microarrays and data processing**

Total RNA was purified from peripheral blood mononuclear cells (PBMCs) of patients with PMF using QIAGEN RNeasy Mini Kit (Qiaqen Cat# 217004) and subjected to the Affymetrix HG-U133Plus2 assay, performed according to the manufacturer’s instructions at the genomic core facility of the Mayo Clinic. The resulting individual mRNA expression profiles for each probe were generated and analyzed using MAPP software:

<http://bioinformatics.mayo.edu/BMI/bin/view/Main/BioinformaticsCore/MAPP>

"Probesets were annotated using current Affymetrix annotation files. Differential expression was determined using a t-test, with the Benjamini–Hochberg method applied to control the false discovery rate (FDR). Genes with an adjusted *P*-value < 0.05 and an absolute fold change >1.5 were considered differentially expressed.

**Myeloproliferative neoplasm (MPN) patient samples**

The current study was approved by the Mayo Clinic Institutional Review Board and conducted in accordance with the Declaration of Helsinki. The diagnoses of MPN leukemia were made according to the criteria of World Health Organization. Mononuclear cells (MNC; >70% of blasts) from PB of untreated PMF patients were obtained through the Mayo Clinic Rochester Leukemia Tissue Bank. For BMS-911543 therapy, the details were described in our recent publication.[[3](#_ENREF_3)] PB cells were directly used for molecular biological assays without further cell culture. All patients signed an informed consent document approved by the Institutional Review Board before entering any study.

**Animal studies**

C57BL/6 mice (female, 4-6 weeks old) were purchased from The Jackson Laboratory (Bar Harbor, ME). All animal experiments were approved by the Institutional Animal Care and Use Committees of the University of Minnesota and were in accordance with the U.S. National Institutes of Health (NIH) Guide for Care and Use of Laboratory Animals. A total of 2 × 10^6^ HEL cells in 100 μl of PBS were injected subcutaneously into the flanks of 6-week-old nude mice. Tumor diameters will be measured after 5 days from injection and then every 3 days. When tumor size approached approximately 30 mm^3^, the animals 1) received 5 mg/kg of MLN9708 in PEG400 and saline (ratio 15:38:47) or vehicle alone (intravenous bolus), three times each week for two weeks, or 2) the animals received 16 mg/kg of emetine or vehicle alone (intravenous bolus), three times each week for two weeks. End point tumor sizes were analyzed by using the formula π/6×A×B×C, where A is the length, B is the width, C is the height and expressed in cubic millimeter (mm^3^).[[1](#_ENREF_1),[2](#_ENREF_2),[4](#_ENREF_4)] Mice were sacrificed after 20 days, and the tumors in both control and treatment groups will be collected for further investigations, including H&E and IHC staining. One-half of the tumor tissue from each mouse was collected and frozen immediately in liquid nitrogen and then transferred to a -80°C freezer for subsequent molecular analysis. The other half of each tumor was fixed in 10% neutral buffered formalin for 24 hours at 4°C and then transferred to phosphate buffered saline to make paraffin embedded blocks for H&E and IHC staining.

**Statistical analysis**

Sample sizes for each study were determined based on literature documentation of similar well-characterized experiments to ensure sufficient power for statistical analysis of experimental vs control outcomes. *In vitro* experiments (e.g., qPCR, cell proliferation assays, dot blotting, clonogenic assays) were routinely repeated three times unless otherwise indicated in the figure legends or main text. Each reported value represents the mean ± SEM of at least three independent experiments. No samples or animals were excluded from the analysis. All criteria were pre-established. No randomization was used in our studies. No blinding for all experiments. The statistical tests were justified as appropriate for every figure. All data meets the normality assumptions of the tests. Variations were compatible between groups.

All statistical analyses were performed using GraphPad Prism software. Data were compared using the two-tailed Student’s t-test. Tissue array, Western Blot, real-time PCR, dot-blot, tumor growth, tumor weight, and spleen weight data were analyzed using the Student's t-test or one-way ANOVA, as appropriate. Correlation data were analyzed using Pearson correlation coefficients. Differences were considered statistically significant at *P* < 0.05. All *P* values were two-sided.

**SUPPLEMENTARY References**

1. Gao XN, Yan F, Lin J, Gao L, Lu XL, Wei SC*, et al.* AML1/ETO cooperates with HIF1alpha to promote leukemogenesis through DNMT3a transactivation. Leukemia **2015**

2. Shen N, Yan F, Pang J, Wu LC, Al-Kali A, Litzow MR*, et al.* A nucleolin-DNMT1 regulatory axis in acute myeloid leukemogenesis. Oncotarget **2014**;5:5494-509

3. Gangat N, Caramazza D, Vaidya R, George G, Begna K, Schwager S*, et al.* DIPSS plus: a refined Dynamic International Prognostic Scoring System for primary myelofibrosis that incorporates prognostic information from karyotype, platelet count, and transfusion status. Journal of clinical oncology : official journal of the American Society of Clinical Oncology **2011**;29:392-7

4. Yan F, Shen N, Pang J, Xie D, Deng B, Molina JR*, et al.* Restoration of miR-101 suppresses lung tumorigenesis through inhibition of DNMT3a-dependent DNA methylation. Cell death & disease **2014**;5:e1413
